# Simulation of the Electrochemical Impedance in a Three-Dimensional, Complex Microstructure of Solid Oxide Fuel Cell Cathode and Its Application in the Microstructure Characterization

**DOI:** 10.3389/fchem.2021.627699

**Published:** 2021-05-27

**Authors:** Vishwas Goel, Dalton Cox, Scott A. Barnett, Katsuyo Thornton

**Affiliations:** ^1^ Department of Materials Science and Engineering, University of Michigan, Ann Arbor, MI, United States; ^2^ Department of Materials Science and Engineering, Northwestern University, Evanston, IL, United States

**Keywords:** electrochemical impedance spectroscopy (EIS), tortuosity, 3D microstructure, solid oxide fuel cells, Adler-Lane-Steele model, Gerischer impedance

## Abstract

Electrochemical impedance spectroscopy (EIS) is a powerful technique for material characterization and diagnosis of the solid oxide fuel cells (SOFC) as it enables separation of different phenomena such as bulk diffusion and surface reaction that occur simultaneously in the SOFC. In this work, we simulate the electrochemical impedance in an experimentally determined, three-dimensional (3D) microstructure of a mixed ion-electron conducting (MIEC) SOFC cathode. We determine the impedance response by solving the mass conservation equation in the cathode under the conditions of an AC load across the cathode’s thickness and surface reaction at the pore/solid interface. Our simulation results reveal a need for modifying the Adler-Lane-Steele model, which is widely used for fitting the impedance behavior of a MIEC cathode, to account for the difference in the oscillation amplitudes of the oxygen vacancy concentration at the pore/solid interface and within the solid bulk. Moreover, our results demonstrate that the effective tortuosity is dependent on the frequency of the applied AC load as well as the material properties, and thus the prevalent practice of treating tortuosity as a constant for a given cathode should be revised. Finally, we propose a method of determining the aforementioned dependence of tortuosity on material properties and frequency by using the EIS data.

## Introduction

With rising CO_2_ levels in the atmosphere, low emission energy technologies for energy conversion and storage are needed to mitigate further increases in the global temperature. Such technologies in active research and development include, Li-ion batteries (Nitta et al., 2015), supercapacitors (Zhang L. et al., 2018), and several types of fuel cells like polymer electrolyte membrane fuel cells (PEMFC) (Wang et al., 2020), solid acid fuel cells (SAFC) (Lim et al., 2020), biofuel cells (BFC) (Shakeel et al., 2019), and solid oxide fuel cells (SOFC) (Mahato et al., 2015). In particular, SOFCs can be used to produce electricity or, when used in reverse (as a solid oxide electrolyzer cell), to produce fuel, depending upon their applied polarization. Specifically, anodically polarized cells act as low-emission fuel-flexible electrochemical engines, and cathodically polarized cells store energy in the form of stable chemical bonds in H_2_, CO, or CH_4_ through electrolysis (Chen and Jiang, 2016; Mogensen et al., 2019). However, conventional high temperature SOFCs run at 800–1000°C, which makes their operation and maintenance highly cost-ineffective (Gao et al., 2016). The operating temperature has been reduced by the use of electrocatalytically active mixed ion-electron conducting (MIEC) cathodes such as (La,Sr) (Co,Fe)O_3–δ_ (LSCF) (Hwang et al., 2005; Liu et al., 2013; Niania et al., 2020) and Sr(Ti,Fe)O_3–δ_ (STF) (Yoo and Bouwmeester, 2012; Perry et al., 2015; Nenning et al., 2017), wherein the oxygen evolution reactions (OERs) and oxygen reduction reactions (ORRs) occur over the entire surface. To take advantage of this behavior, SOFC cathodes are designed to have complex and porous microstructures with a large specific surface area; however, such designs result in a high degree of tortuosity for ion transport, which limits the performance of SOFCs. Thus, it is important to accurately determine the tortuosity of a cathode microstructure and optimize it to enable high performance at low operating temperatures.

Several methods have been reported in the literature for determining the tortuosity of a porous electrode. These methods include: a) porosity-tortuosity relations, such as Bruggeman’s relations (Bruggeman, 1935); b) calculations of the tortuous path length either through the distance propagation method (Chen-Wiegart et al., 2014) or the shortest path search method (Çeçen et al., 2012); and c) calculations of tortuosity based on the effective diffusivity (Wilson et al., 2006). Bruggeman’s relations are known to be less suitable for a domain with connected solid phases and complex porous networks (as in a MIEC cathode) (Tjaden et al., 2018). The methods involving the calculations of tortuous path length or the effective diffusivity require the three-dimensional microstructural data as input, which is obtained through tomography (Wilson et al., 2006; Cooper et al., 2016). Unfortunately, availability of the necessary equipment to obtain tomographic data is not ubiquitous, sample preparation is time consuming, and the calculations are computationally expensive. Furthermore, these approaches provide tortuosity values associated with steady-state diffusion, yielding a single value and do not generally describe the tortuosity relevant to oscillating load or when surface reaction is present. Therefore, there is a need for alternate methods for determining tortuosity of a given SOFC electrode. Yu et al. (Yu et al., 2020) reported that tortuosity can be extracted from the diffusional impedance data; in particular, they showed that in systems limited by only bulk diffusion, the diffusional impedance data can be used to characterize a given microstructure in terms of tortuosity and the area of the loading boundary. They also showed that tortuosity is a function of the AC load frequency, which they define as effective tortuosity. Such use of impedance data to characterize the microstructure of a SOFC cathode is valuable to SOFC researchers since they widely use electrochemical impedance spectroscopy (EIS) to test SOFC performance.

In this study, we show that the EIS data of a SOFC MIEC cathode can be used to determine the tortuosity of the solid phase within the cathode. In particular, we consider a statistically representative (Scott Cronin et al., 2012) portion of an experimentally determined complex three-dimensional microstructure of an unbiased SOFC cathode. In the microstructure, we solve for the amplitude of the concentration-response to an applied AC load under the influence of surface reaction and bulk diffusion, and subsequently, use the solution to determine the impedance behavior. We investigate the effect of different material properties (bulk diffusion coefficient and reaction rate constant) on the impedance behavior, and on the effective tortuosity of the microstructure. From our simulations, we find that, due to the presence of surface reaction, the amplitude of oscillations in the oxygen vacancy concentration at the pore/solid interface of the cathode is lower than the amplitude within the bulk of the solid phase. Moreover, the difference between the two concentration amplitudes increases with an increase in the ratio between the reaction rate constant and the bulk diffusion coefficient. Such a difference is not taken into account by the widely used Adler-Lane-Steele (ALS) model, a macrohomogeneous model developed by Adler, Lane and Steele to predict the impedance data of a SOFC MIEC cathode (Adler et al., 1996; Adler, 1998). Thus, we propose a modification to the ALS model to account for the aforementioned difference.

Furthermore, we develop a method of extracting the effective tortuosity from the impedance data. Specifically, we compare the macrohomogeneous (modified ALS) and 1D Finite Length Gerischer relations for the impedance to determine the effective tortuosity. Our calculations for tortuosity determination reveal that the effective tortuosity is a function of the microstructure, frequency of the applied AC load, and the material properties such as the reaction rate constant and bulk diffusion constant. Additionally, the effective tortuosity of a microstructure in the low-frequency regime (where the tortuosity approaches the DC value) decreases with an increase in the ratio between the reaction rate constant and the bulk diffusion coefficient due to a decreased penetration depth of the electrode reactions. This finding suggests that the prevalent practice of using a single tortuosity value for a given electrode for extracting the material properties by employing the ALS model (Adler, 1998; Zhang S.-L. et al., 2018; Zhang S.-L. et al., 2019) should be reviewed and revised. Additionally, the finding suggests that, both the intrinsic material properties and the microstructure should be considered concurrently in designing a cathode with enhanced performance. Our findings open a new array of applications for the EIS technique in characterizing and optimization of the microstructure of a SOFC cathode.

All the previous reports primarily consider artificially generated microstructures (Rüger et al., 2007; Haffelin et al., 2013; Pereira et al., 2014), which are designed to match given macrohomogeneous properties and thus may not be representative of the local microstructural morphologies and topologies. Only a few three-dimensional impedance calculations have been reported with a consideration of experimentally determined microstructures that are statistically representative of the cathode (Kreller et al., 2011; Lynch et al., 2013). The report by Kreller et al. (Kreller et al., 2011) only explicitly considered the three-dimensional microstructure near the electrode/electrolyte interface and assumed a one-dimensional macrohomogeneous domain beyond a certain distance from the interface. On the other hand, the investigation by Lynch et al. (Lynch et al., 2013) focused on the method of calculating impedance in a microstructure where the ORR occurs either through bulk or surface pathways. However, it did not propose the utilization of the calculated impedance data for characterizing the microstructure nor did it consider the difference between the concentration amplitudes at the pore/solid interface and within the bulk of the solid, which are the two focuses of this paper.

## Methods

### Model Equations for Impedance Calculation

**FIGURE 1:**
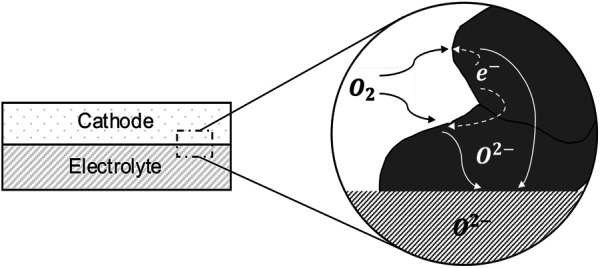
A schematic of oxygen transport withing the solid phase (gray region) of a MIEC SOFC cathode; the schematic is based on Kreller, (Kreller, 2011).

MIEC cathodes are known to have much higher electronic conductivity as compared to the ionic conductivity (Jung and Tuller, 2009); thus, we ignore any impedance contribution due to electronic resistance. Moreover, due to the high electronic conductivity, it is reasonable to assume that the electrostatic potential of the solid phase is spatially invariant. Thus, in the absence of any gas phase transport limitation, the cathode impedance can be entirely attributed to the reduction of oxygen at the pore/solid interface and the transport of the resulting oxygen ions through the solid in the cathode, which is shown in Figure 1. The oxygen ions diffuse inside the solid phase of the cathode through a vacancy mechanism. Hence, the ionic current can be calculated from the flux of the oxygen vacancies. The mass transport equation for the oxygen vacancies inside the cathode can be written as

$$\frac{\partial C_{v}}{\partial t}=\nabla \cdot \left(AD_{v}\nabla C_{v}\right)\;\in \Omega ,$$ (1)

where $C_{v}$ represents the concentration of oxygen vacancies, A represents the thermodynamic factor, which is defined by Adler, Lane, and Steele (Adler et al., 1996) as a factor relating the oxygen vacancy concentration to the partial pressure of oxygen gas in the pore phase, $A=\left(1/2\right)\partial \ln\left(P_{O_{2}}\right)/\partial \ln\left(C_{v}\right)$. The diffusivity of the vacancies inside the cathode is denoted by $D_{v}$, whereas t represents time, and Ω represents the solid phase region within the cathode where the vacancy transport takes place. The reaction at the pore/solid interface within the cathode can be modeled as a first-order chemical reaction as

$$-AD_{v}\nabla C_{v}=\kappa^{3D}\left(C_{v}-C_{v}^{0}\right)\;\in \delta \Omega ,$$ (2)

where $\kappa^{3D}$ is the reaction rate constant, $C_{v}^{0}$ is the equilibrium vacancy concentration and δΩ represents the pore/solid interface.

We employ the smoothed boundary method (SBM) (Yu et al., 2012) to solve Eq. 1 within an experimentally determined, complex microstructure of the MIEC cathode, along with the boundary condition set in Eq. 2. Using SBM, these equations can be reformulated as

$$\psi \frac{\partial C_{v}}{\partial t}=\nabla \cdot \left(\mathrm{\psi A}D_{v}\nabla C_{v}\right)-\left|\nabla \psi \right|\kappa^{3D}\left(C_{v}-C_{v}^{0}\right),$$ (3)

where ψ is a domain parameter that represents the geometry of the complex microstructure. Within the pores, ψ has a value of zero, and within the solid phase, it has a value of one. At the pore/solid interface, it smoothly transitions between zero and one. Further details about the process of obtaining ψ for a complex microstructure can be found in our previous work (Yu et al., 2020).

The application of an AC load causes the vacancy concentration, $C_{v}$, to oscillate about $C_{v}^{0}$. The oscillating perturbation is denoted by ΔC, and thus, the relation between $C_{v},C_{v}^{0}$, and ΔC can be written as

$$C_{v}=C_{v}^{0}\mathrm{+\Delta}C.$$ (4)

Since $C_{v}^{0}$ is constant throughout space at a given temperature, the spatial variation of $C_{v}$ can be described entirely in terms of ΔC as

$$\psi \frac{\partial \mathrm{\Delta C}}{\partial t}=\nabla \cdot \left(\mathrm{\psi A}D_{v}\nabla \left(\Delta C\right)\right)-\left|\nabla \psi \right|\kappa^{3D}\Delta C.$$ (5)

Furthermore, $AD_{v}$ can be assumed to be a constant for a MIEC cathode at a given temperature and for a small value of ΔC (Mizusaki et al., 1993). Therefore, Eq. 5 can be written as

$$\psi \frac{\partial \Delta C}{\partial t}=AD_{v}\nabla \cdot \left(\psi \nabla \left(\Delta C\right)\right)-\left|\nabla \psi \right|\kappa^{3D}\Delta C.$$ (6)

Eq. 6 can be further simplified by expressing the time dependent part of the oscillations as complex exponential functions, i.e.,

$$\Delta C=\frac{\tilde{C}e^{\mathrm{i\omega t}}+{\tilde{C}}^{*}e^{-\mathrm{i\omega t}}}{2},$$ (7)

where $\tilde{C}$ is a complex quantity that varies in space with ${\tilde{C}}^{*}$ as its complex conjugate, ω is the frequency of the AC load, and i is the imaginary unit. Let $C_{R}$ and $C_{I}$ be the real and imaginary components of $\tilde{C}$. Upon the substitution of Eq. 7 into Eq. 6, the subsequent collection of real and imaginary terms, and the cancellation of the exponential functions, we obtain

$$AD_{v}\nabla \cdot \left(\psi \nabla C_{R}\right)-\left|\nabla \psi \right|\kappa^{3D}C_{R}\mathrm{=-}\psi \omega C_{I},$$ (8a)

$$AD_{v}\nabla \cdot \left(\psi \nabla C_{I}\right)-\left|\nabla \psi \right|\kappa^{3D}C_{I}=\psi \omega C_{R}.$$ (8b)

The above pair of equations is then solved to determine the $C_{R}$ and $C_{I}$ as functions of space and ω. Once $C_{R}$ and $C_{I}$ are known, the impedance, Z(ω), of the cathode can be calculated as (Jacobsen and West, 1995; Nielsen et al., 2011)

$$Z\left(\omega \right)=\frac{\mathrm{RTL}}{\left(-n^{2}F^{2}D_{v}C_{v}^{0}\right)}\frac{\tilde{C}{|}_{\mathrm{x=}0}/L}{\langle \partial \tilde{C}/\partial x{|}_{\mathrm{x=}0}\rangle },$$ (9)

where R  is the universal gas constant, T is temperature, n is the number of moles of electrons consumed in the reaction, F is Faraday’s constant, L is the thickness of the cathode, and $\langle \partial \tilde{C}/\partial x{|}_{x=0}\rangle$ is the gradient of the concentration amplitude in the primary diffusion direction, x, averaged over the loading boundary located at x =0, which represents the electrolyte/cathode interface. Furthermore, the first term on the right-hand side of Eq. 9 is the product of the material resistivity and thickness of the cathode, and it can be defined as $Z_{0}$. Therefore, Eq. 9 becomes

$$Z\left(\omega \right)=Z_{0}\frac{\tilde{C}{|}_{\mathrm{x=}0}/L}{\langle \partial \tilde{C}/\partial x{|}_{\mathrm{x=}0}\rangle }.$$ (10)

For numerically solving Eqs 8a,b, we chose to make them nondimensional by defining a length scale, l; then, $\hat{\nabla }=l\nabla$. Hereafter, the circumflex $\left(\hat{.}\right)$ symbol denotes that the associated operator or quantity is nondimensional. Substituting $\hat{\nabla }/1$ in place of ∇, and multiplying both sides with $\left(l^{2}/\left(AD_{v}\right)\right)$ gives

$$\hat{\nabla }\cdot \left(\psi \hat{\nabla }C_{R}\right)-\left|\hat{\nabla }\psi \right|\kappa^{3D}\frac{l}{AD_{v}}C_{R}\mathrm{=-}\psi \omega \frac{l^{2}}{AD_{v}}C_{I},$$ (11a)

$$\hat{\nabla }\cdot \left(\psi \hat{\nabla }C_{I}\right)-\left|\hat{\nabla }\psi \right|\kappa^{3D}\frac{l}{AD_{v}}C_{I}=\psi \omega \frac{l^{2}}{AD_{v}}C_{R}.$$ (11b)

By defining ${\hat{\kappa }}^{3D}=\kappa^{3D}l/\left(AD_{v}\right)$ and $\hat{\omega }=\omega l^{2}/\left(AD_{v}\right)$ we obtain

$$\hat{\nabla }\cdot \left(\psi \hat{\nabla }C_{R}\right)-\left|\hat{\nabla }\psi \right|{\hat{\kappa }}^{3D}C_{R}=-\psi \hat{\omega }C_{I},$$ (12a)

$$\hat{\nabla }\cdot \left(\psi \hat{\nabla }C_{I}\right)-\left|\hat{\nabla }\psi \right|{\hat{\kappa }}^{\mathrm{3D}}C_{I}=\psi \hat{\omega }C_{R},$$ (12b)

which are solved for two different sets of boundary conditions (BCs) as listed in Table 1.

**TABLE 1:** Sets of boundary conditions applied to the system of equations, Eq.12a,12b.

Set of BCs\Location	$\hat{x}0$	$\hat{x}\hat{L}$
Blocking current collector (BCC) BC	$C_{R}=1$, $C_{I}=0$	$\partial C_{R}/\partial \hat{x}=0$, $\partial C_{I}/\partial \hat{x}=0$
Transmissive current collector (TCC) BC	$C_{R}=1$, $C_{I}=0$	$C_{R}=0$, $C_{I}=0$

The two sets of the BCs differ only in those at the cathode/current collector interface $\left(\hat{x}=\hat{L}\right)$, as shown in Table 1. The blocking current collector (BCC) BC represents the case of a foil-like current collector, which blocks all the ionic current at the cathode/current collector interface. This condition is more representative of the SOFC cathodes and is also used by the ALS model (Adler et al., 1996). However, in some reports (Boukamp et al., 2003; Boukamp et al., 2006; Holtappels et al., 2006) the impedance behavior of MIEC oxides has also been fit with the relation that is applicable to the transmissive boundary condition. Under this boundary condition, the vacancy concentration is set to be zero at the cathode/current collector interface, and we will refer to this as transmissive current collector (TCC) BC, which may correspond to a porous or mixed conducting current collector. We simulate the impedance behavior for both of these sets of boundary conditions to cover all the scenarios reported in the literature. The results for TCC BC are provided in Supplementary Material. Moreover, a list of all the variables and symbols used in this article is provided in Table 2.

**TABLE 2:** List and description of the variables and symbols used in the model equations.

Symbol	Description
$C_{v}$	Concentration of oxygen vacancies
A	The thermodynamic factor relating the oxygen vacancy concentration to the partial pressure of oxygen gas in the pore phase, $A=\left(1/2\right)\partial \ln\left(P_{O_{2}}\right)/\partial \ln\left(C_{v}\right)$
$D_{v}$	Diffusivity of the oxygen vacancies within the solid bulk
Ω	Solid phase region within the cathode
δΩ	Pore/solid interface
$\kappa^{3D}$	Reaction rate constant
ψ	Domain parameter that represents the geometry of the complex microstructure. Within the pores, ψ has a value of zero, and within the solid phase, it has a value of one
ΔC	Perturbation in the oxygen vacancy concentration caused by the applied AC load
$\tilde{C}$	Complex quantity that varies in space
$\tilde{C}*$	Complex conjugate of $\tilde{C}$
ω	Frequency of the AC load
i	Imaginary unit
$C_{R}$	Real component of $\tilde{C}$
$C_{I}$	Imaginary component of $\tilde{C}$
Z(ω)	Impedance
R	Universal gas constant
T	Temperature
n	Number of moles of electrons consumed in the electrochemical reaction
F	Faraday’s constant
L	Thickness of the cathode
$Z_{0}$	Product of the material resistivity and thickness of the cathode
$\left(\;\hat{.}\right)$	The circumflex symbol denotes that the associated operator or quantity is nondimensional
$Z^{3D}$	Impedance of a finitely thick MIEC SOFC cathode
$R_{chem}$	Characteristic resistance describing the chemical contributions to the cathode impedance; as defined in the ALS model
$t_{\mathrm{chem}}$	Relaxation time related to the chemical processes of solid-state diffusion and oxygen surface exchange; as defined in the ALS model
δ	Characteristic distance related to the chemical processes of solid-state diffusion and oxygen surface exchange; as defined in the ALS model
ϵ	The microstructure porosity
τ	The microstructure tortuosity
κ	Surface exchange coefficient; as defined in the ALS model
a	Interfacial surface area per unit cathode volume
$C_{\mathrm{mc}}$	The oxygen site concentration in the mixed conductor (mc)
$\kappa^{1D}$	Macrohomogeneous reaction rate constant
$Z^{\mathrm{FLG}}$	The FLG impedance
$C_{v}^{s}$	Oxygen vacancy concentration at the pore/solid interface
$\Delta C^{s}$	The concentration oscillation at the pore/solid interface
α	The ratio between the interface and the bulk concentration oscillations
〈*α*〉	Volume averaged α
$\langle \alpha_{0}\rangle$	DC value of 〈α〉

### Numerical Implementation

The choice of SBM enabled the use of a standard Cartesian grid and the finite difference method to solve the above pair of equations (Eqs 12a,b). We used the center difference scheme to discretize the domain, which consists of 352 × 642 × 594 grid points. A uniform grid spacing (h = 0.0272, non-dimensional) was selected to ensure the presence of at least 3 grid points across the pore/solid interface (λ= 0.0817). The selection of the numerical parameters is discussed in Supplementary Material in detail. The Alternating-Direction Line-Relaxation (ADLR) method (Hofhaus and Van de Velde, 1996) was employed to solve the equations. The ADLR method utilizes a tridiagonal matrix solve to obtain the values of the solutions individually along the x, y, and z directions. To simultaneously solve Eqs 12a,b, an iterative scheme was developed, in which the ADLR method was first used to calculate $C_{R}$ for a given RHS of Eq. 12a, and the resulting values were then used to update the RHS of Eq. 12b. Subsequently, $C_{I}$ was obtained using Eq. 12b with the updated value of $C_{R}$. This procedure was repeated until both $C_{R}$ and $C_{I}$ numerically converged. We defined the convergence metric as the ratio between the absolute value of the sum of all elements of the change matrix (obtained by taking the difference between the updated concentration and previous concentration) and the sum of all elements of the old concentration matrix. The solution was deemed converged when the metric became less than a specified threshold value. We deduced the value by progressively reducing the value and observing the change in the resulting solution. We found that solution did not change appreciably between the threshold values of $10^{\mathrm{-10}}$ and $10^{\mathrm{-11}}$. Thus, we selected a value of $10^{\mathrm{-10}}$ as the criteria the numerical convergence of our solution.

We chose l = 0.46  μm as the length scale for the non-dimensionalization. The reader is referred to our previous work (Yu et al., 2020) for more details on the selection of l. Finally, a wide range of values for $AD_{v}$ and $\kappa^{3D}$ are reported in the literature (Zhang L. et al., 2018) because of the use of different cathode materials and operating temperatures (600–800°C). We chose three values of ${\hat{\kappa }}^{3D}$ for our study in order to cover this wide range and simulated the impedance behavior for the frequency values, $\hat{\omega }$, between 0 and 2.672. It should be noted that the value of ${\hat{\kappa }}^{3D}$ is affected by the values of both $AD_{v}$ and $\kappa^{3D}$.

### Derivation for Impedance Expressions and Transcendental Equation for Tortuosity

Adler, Lane, and Steele proposed a macrohomogeneous model to predict the impedance, $Z^{3D}$, of a finitely thick MIEC SOFC cathode, which is commonly known as the ALS model (Adler et al., 1996; Adler, 1998). The model gives the impedance response for a symmetric cell with two identical cathodes. Since we consider a half cell with a single cathode in our investigation, the ALS impedance expression is divided by 2. Thus,

$$Z^{3D}=\frac{R_{\mathrm{chem}}}{\sqrt{\left(\mathrm{1+}\mathrm{i\omega}t_{\mathrm{chem}}\right)}}\coth\left(\frac{L}{\delta }\sqrt{\left(\mathrm{1+}\mathrm{i\omega}t_{\mathrm{chem}}\right)}\right),$$ (13a)

$$R_{\mathrm{chem}}=\frac{\mathrm{RT}}{4F^{2}}\sqrt{\left(\frac{\tau^{2}}{\left(\mathrm{1-ɛ}\right)C_{v}^{0}D_{v}\mathrm{a\kappa}C_{\mathrm{mc}}}\right)},$$ (13b)

$$t_{\mathrm{chem}}=\frac{C_{v}^{0}\left(\mathrm{1-ɛ}\right)}{\mathrm{Aa\kappa}C_{\mathrm{mc}}},$$ (13c)

$$\delta =\sqrt{\left(\frac{C_{v}^{0}D_{v}\left(\mathrm{1-}ɛ\right)}{a\tau^{2}\kappa C_{\mathrm{mc}}}\right)},$$ (13d)

where τ is the microstructure tortuosity, ɛ is the microstructure porosity, κ is the surface exchange coefficient, a is interfacial surface area per unit cathode volume, $\;C_{mc}$ is the oxygen site concentration (in the unit of mol/m^3^) in the mixed conductor (mc). The ALS model defines the reaction rate as $\mathrm{A\kappa}C_{\mathrm{mc}}\Delta C/C_{v}^{0}$, whereas we define it as $\kappa^{3D}\Delta C$ (Eq. 2). Therefore, κ and $\kappa^{3D}$ are related as

$$\kappa^{\mathrm{3D}}=\frac{\mathrm{A\kappa}C_{\mathrm{mc}}}{C_{v}^{0}}.$$ (14)

It should be noted that in the original ALS expression, τ is used for denoting tortuosity factor, whereas in this article we use τ for denoting tortuosity. These two quantities are related; the tortuosity factor is the square of the tortuosity.

The substitution of Eqs 13b,c,d, and Eq. 14 into Eq. 13a gives

$$Z^{\mathrm{3D}}=\frac{\mathrm{RTL}}{4F^{2}C_{v}^{0}D_{v}}\frac{\tau^{2}}{\mathrm{1-ɛ}}\frac{\coth\left(\mathrm{\tau L}\sqrt{\frac{\frac{a\kappa^{\mathrm{3D}}}{\mathrm{1-ɛ}}\mathrm{+i\omega}}{AD_{v}}}\right)}{\mathrm{\tau L}\sqrt{\frac{\frac{a\kappa^{\mathrm{3D}}}{\mathrm{1-ɛ}}\mathrm{+i\omega}}{AD_{v}}}}.$$ (15)

By defining $\kappa^{1D}=\kappa^{3D}a/\left(\mathrm{1-}ɛ\right)$ as the macrohomogeneous reaction rate constant and by using the definition for $Z_{0}$ we obtain

$$Z^{\mathrm{3D}}=Z_{0}\frac{\tau^{2}}{\mathrm{1-\varepsilon}}\frac{\coth\left(\mathrm{\tau L}\sqrt{\frac{\kappa^{\mathrm{1D}}\mathrm{+i\omega}}{AD_{v}}}\right)}{\mathrm{\tau L}\sqrt{\frac{\kappa^{\mathrm{1D}}\mathrm{+i\omega}}{AD_{v}}}}.$$ (16)

Finally, by following the same methodology as described in the model equation section, Eq. 16 can be made nondimensional as

$$\frac{Z^{\mathrm{3D}}}{Z_{0}}=\frac{\tau^{2}}{\mathrm{1-\varepsilon}}\frac{\coth\left(\tau \hat{L}\sqrt{{\hat{\kappa }}^{\mathrm{1D}}+i\hat{\omega }}\right)}{\tau \hat{L}\sqrt{{\hat{\kappa }}^{\mathrm{1D}}+i\hat{\omega }}},$$ (17)

where the nondimensional quantities are defined as $\hat{L}=L/l$, ${\hat{\kappa }}^{1D}=\kappa^{1D}l^{2}/\left(AD_{v}\right)$, and $\hat{\omega }=\omega l^{2}/\left(AD_{v}\right)$.

The conversion of Eq. 13 into Eq. 17 enables a direct comparison of the impedance expression of a complex 3D microstructure with that of the standard expression for 1D Finite Length Gerischer (FLG) impedance. The 1D FLG element represents the impedance of a 1D MIEC domain where no microstructural effects are present and where the kinetics is co-limited by both surface reaction and bulk diffusion. The FLG impedance, $Z^{\mathrm{FLG}}$ for a 1D domain with the same macrohomogeneous reaction rate constant, ${\hat{\kappa }}^{1D}$, and thickness, $\hat{L}$, as the SOFC cathode under consideration can be written as

$$\frac{Z^{\mathrm{FLG}}}{Z_{0}}=\frac{\coth\left(\hat{L}\sqrt{{\hat{\kappa }}^{\mathrm{1D}}+i\hat{\omega }}\right)}{\hat{L}\sqrt{{\hat{\kappa }}^{\mathrm{1D}}+i\hat{\omega }}},$$ (18)

It is evident from a comparison between Eq. 17 and Eq. 18 that the microstructure affects the impedance response of a SOFC cathode in two ways. First, the microstructure increases the cathode impedance by a factor of $\tau^{2}/\left(\mathrm{1-}ɛ\right)$. Second, the effective thickness of the cathode is increased by a factor of τ. These effects can be exploited in the following way to determine the value of τ using the value of $Z^{3D}$, which is experimentally known.

A ratio between Eq. 17 and Eq. 18 gives

$$\frac{Z^{\mathrm{3D}}}{Z^{\mathrm{FLG}}}=\frac{\tau }{\mathrm{1-ɛ}}\frac{\coth\left(\tau \hat{L}\sqrt{{\hat{\kappa }}^{\mathrm{1D}}+i\hat{\omega }}\right)}{\coth(\hat{L}\sqrt{{\hat{\kappa }}^{\mathrm{1D}}+i\hat{\omega }})}.$$ (19)

By taking the modulus (which is analytically unnecessary but numerically required) and after some rearrangement, Eq. 19 can be written as

$$\left|\mathrm{\tau coth}\left(\tau \hat{L}\sqrt{{\hat{\kappa }}^{\mathrm{1D}}+i\hat{\omega }}\right)\right|=\left|\frac{Z^{\mathrm{3D}}}{Z^{\mathrm{FLG}}}\left(\left(\mathrm{1-ɛ}\right)\coth\left(\hat{L}\sqrt{{\hat{\kappa }}^{\mathrm{1D}}+i\hat{\omega }}\right)\right)\right|$$ (20)

where terms containing τ are collected on the left-hand side of the equation, yielding a transcendental equation for τ. The nondimensional macrohomogeneous reaction rate constant, ${\hat{\kappa }}^{1D}=\kappa^{1D}l^{2}/\left(AD_{v}\right)=al^{2}\kappa^{3D}/\left(\left(\mathrm{1-}ɛ\right)AD_{v}\right)$, is dependent on material properties, namely $\kappa^{3D}$ and $AD_{v}$, which can be determined from the literature for standard materials, as well as on microstructural characteristics, namely a and ϵ, both of which can be determined experimentally (e.g., using Mercury intrusion porosimeter (Rootare and Prenzlow, 1967). Therefore, with both ${\hat{\kappa }}^{1D}$ and $Z^{3D}$ known, Eq. 20 can be solved to determine the effective tortuosity. However, before implementing this method, we need to make a modification to the ALS model as described in the following section.

## Simulation Results and Discussion

### Modification to the ALS Model

The ALS model (Adler et al., 1996; Adler, 1998) is derived using the volume averaged form of the mass conservation equation (Eqs 2 and 3), which can be written as

$$\left(\mathrm{1-}\varepsilon \right)\frac{\partial C_{v}}{\partial t}=\frac{AD_{v}\left(\mathrm{1-}\varepsilon \right)}{\tau^{2}}\frac{\partial^{2}C_{v}}{\partial x^{2}}-a\kappa^{3D}\left(C_{v}^{s}-C_{v}^{0}\right).$$ (21)

where $C_{v}^{s}$ is the oxygen vacancy concentration at the pore/solid interface. Eq. 21 can be written in term of ΔC by using the relation described by Eq. 4 as

$$\left(\mathrm{1-ɛ}\right)\frac{\partial \Delta C}{\partial t}=\frac{AD_{v}\left(\mathrm{1-\varepsilon}\right)}{\tau^{2}}\frac{\partial^{2}\Delta C}{\partial x^{2}}\mathrm{-a}\kappa^{\mathrm{3D}}\Delta C^{s}.$$ (22)

**FIGURE 2:**
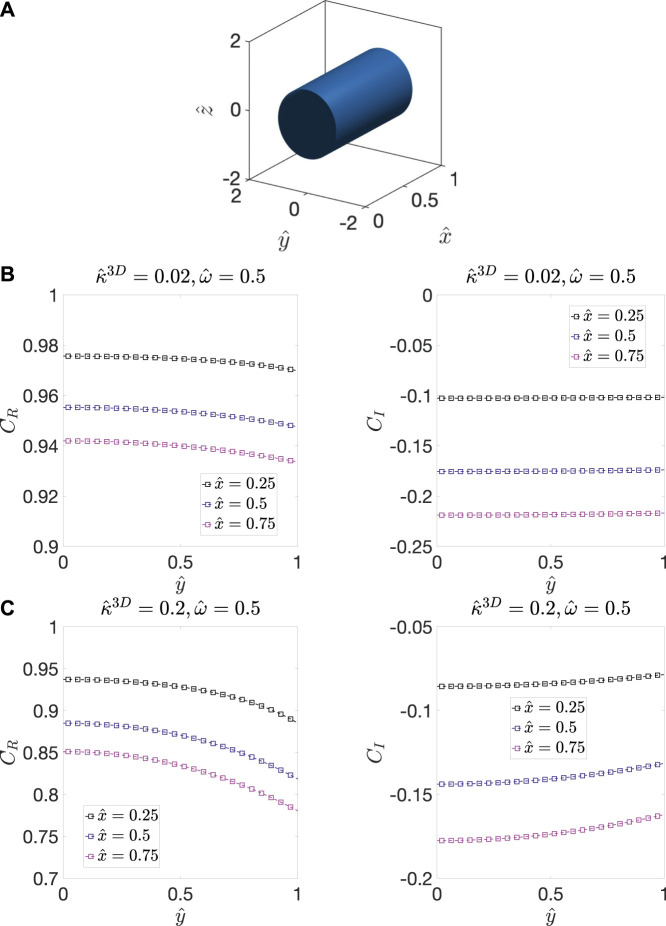
**(A)** The model microstructure, which contains a cylindrical solid domain with unit radius and length surrounded by pore phase. The distribution of $C_{R}$ and $C_{I}$ along the radial axis of the cylinder at different $\hat{x}$ positions for $\hat{\omega }\mathrm{=0}\mathrm{.5}$ for **(B)** ${\hat{\kappa }}^{3D}\mathrm{=0}\mathrm{.02}$, and **(C)**  0.2. The solution values outside the cylinder $\left(\left|\hat{y}\right|\mathrm{>1}\right)$ have no physical meaning as the domain parameter, ψ, is zero there, and therefore are not included in the plots. Due to the radial symmetry of the cylinder, results are only shown for $\hat{y}$ between zero and one.

The ALS model assumes the concentration oscillations at the pore/solid interface, $\Delta C^{s}$, and within the bulk of the solid, ΔC, to be equal. However, in reality, the amplitude of the concentration oscillation, $\tilde{C}$, is smaller at the interface than the amplitude within the bulk because of the surface reaction. We demonstrate this phenomenon by considering a model microstructure with solid cylindrical domain surrounded by the pore phase, as shown in Figure 2A. The dimensions of the microstructure are also shown in the figure, and the primary diffusion direction of the model microstructure is along the x-axis. The reaction occurs at the pore/solid interface, i.e., at the surface of the cylinder. Using this model geometry, we determine $C_{R}$ and $C_{I}$ within the cylinder for $\hat{\omega }\;=0.5$ and for two different values of ${\hat{\kappa }}^{3D}$ by solving Eqs 12a, 12b, which are subject to the BBC BC at $\hat{x}=1$. Figures 2B,C show the distribution of $C_{R}$ and $C_{I}$ along the radius of the cylinder at different positions along the x-axis for ${\hat{\kappa }}^{3D}\mathrm{=0}\mathrm{.02}$ and ${\hat{\kappa }}^{3D}\mathrm{=0}\mathrm{.2}$, respectively. The solution values outside the cylinder $\left(\left|\hat{y}\right|\mathrm{>1}\right)$ have no physical meaning as the domain parameter, ψ, is zero there, and therefore are not included in the plots. As can be seen, for a small value of ${\hat{\kappa }}^{3D}$, 0.02, the magnitude of $C_{R}$ at $\hat{y}=0$ and $\hat{y}=1$ are similar (Figure 2B), whereas for a larger value of ${\hat{\kappa }}^{3D}$, 0.2, the magnitude of $C_{R}$ is significantly smaller at $\hat{y}=1$ than at $\hat{y}=0$, as shown in Figure 2C. The larger extent of spatial variation in $C_{R}$ for higher ${\hat{\kappa }}^{3D}$ is caused by the greater magnitude of $\hat{\nabla }C_{R}$ at the interface, which is directly proportional to ${\hat{\kappa }}^{3D}$. In other words, a large ${\hat{\kappa }}^{3D}$ suggests that the rate of diffusion is slower than the reaction rate, which causes $C_{R}$ to be smaller in magnitude at the interface than within the bulk. This spatial variation is observed throughout the cylinder, and a similar trend is seen for $C_{I}$ as well.

It is evident from the above model case that the difference between the amplitude of the oscillations at the interface and within the bulk cannot be neglected. Thus, we now consider a modification to the ALS model. Let α be the ratio between the interface and the bulk concentration oscillations, i.e., $\mathrm{\alpha =}\Delta C^{s}/\Delta C$. Therefore, Eq. 22 becomes

$$\left(\mathrm{1-ɛ}\right)\frac{\partial \Delta C}{\partial t}=\frac{AD_{v}\left(\mathrm{1-ɛ}\right)}{\tau^{2}}\frac{\partial^{2}\Delta C}{\partial x^{2}}\mathrm{-a}\kappa^{\mathrm{3D}}\left(\alpha \Delta C\right).$$ (23)

Eq. 23 suggests that the effect of α can be incorporated into the ALS model by appropriately modifying ${\hat{\kappa }}^{3D}$. However, this cannot be done as straightforwardly because 〈*α*〉 varies in space. Nonetheless, the complexity can be reduced by considering the volume averaged α, or 〈*α*〉, to modify the value of $\kappa^{3D}$. The quantity 〈α〉 is defined as the ratio between average interface and average bulk concentration amplitudes, i.e.,

$$\langle \alpha \rangle =\frac{\int \tilde{C}\left|\hat{\nabla }\psi \right|d\hat{V}}{\int \left|\hat{\nabla }\psi \right|d\hat{V}}.{\left(\frac{\int \tilde{C}\psi \;d\hat{V}}{\int \psi d\hat{V}}\right)}^{\mathrm{-1}},$$ (24)

where the integral is over the entire volume of the cathode microstructure.

**FIGURE 3:**
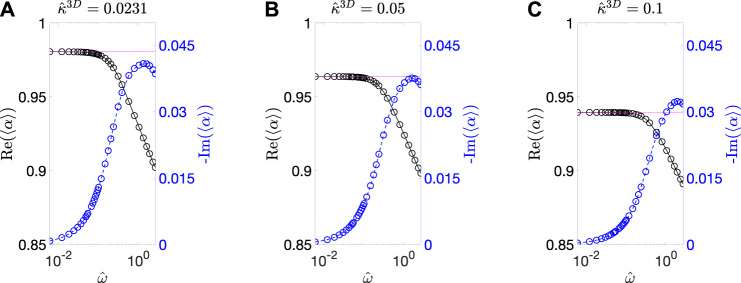
Calculated 〈α〉 vs. $\hat{\omega }$ (BCC BC) at **(A)** ${\hat{\kappa }}^{3D}\;=\;0.0231$, **(B)** 0.05, and **(C)** 0.1. The real component (black solid curve) and the imaginary component (blue dashed curve) of 〈α〉 are plotted on the left and right *y*-axes, respectively. The circles indicate the calculated data point. The DC values of 〈α〉 are shown with horizontal magenta dotted lines as a reference.

**FIGURE 4:**
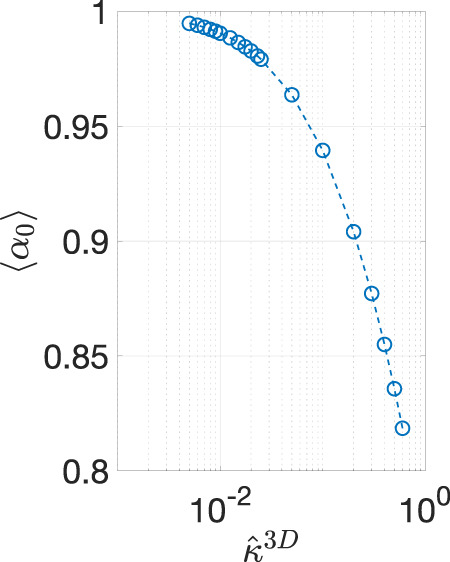
DC value of 〈α〉, 〈$\alpha_{0}$〉, as a function of ${\hat{\kappa }}^{3D}$.

To characterize 〈α〉, we first determined its dependence on $\hat{\omega }$ for the three values of ${\hat{\kappa }}^{3D}$. The results are summarized in Figure 3. Note that examination of the numerical accuracy is presented in Supplementary Material, and it has been shown that the error in the results is within 2%. It can be seen that, for a particular value of ${\hat{\kappa }}^{3D}$, 〈α〉 has much higher real component than the imaginary component at all $\hat{\omega }$ values. Moreover, the real component remains constant with increasing $\hat{\omega }$ before transitioning into a regime where it decreases with increasing $\hat{\omega }$. The $\hat{\omega }$ value where this transition occurs increases with the value of ${\hat{\kappa }}^{3D}$. Furthermore, since 〈α〉 remains constant for a fairly wide range of frequency values, we made a further simplifying assumption by approximating 〈α〉 by its DC value, 〈$\alpha_{0}$〉. We calculated $\langle \alpha_{0}\rangle$ for 19 values of ${\hat{\kappa }}^{3D}$ ranging from $5\times 10^{-3}$ to $6\times 10^{-1}\;$ and summarized the results in Figure 4. The value of 〈$\alpha_{0}\rangle \approx 1$ for small values of ${\hat{\kappa }}^{3D}$, and it monotonically decreases with increasing ${\hat{\kappa }}^{3D}$. Both of these characteristics are expected because small values of ${\hat{\kappa }}^{3D}$ suggest that the reaction rate is lower than the diffusion rate, and thus there is little difference between the interface and bulk concentration amplitudes. However, for large values of ${\hat{\kappa }}^{3D}$, the reaction rate is much larger than the diffusion rate, which causes the concentration amplitude at the pore/solid interface to be lower than the bulk.

Using 〈$\alpha_{0}$〉 as a function of ${\hat{\kappa }}^{3D}$, to capture the difference between the surface and bulk concentration amplitudes, we propose a modified macrohomogeneous reaction rate constant, ${\hat{\kappa }}^{1D}$, in the ALS expression as

$$\frac{Z^{\mathrm{3D}}}{Z_{0}}=\frac{\tau^{2}}{\mathrm{1-\varepsilon}}\frac{\coth\left(\tau \hat{L}\sqrt{\langle \alpha_{0}\rangle {\hat{\kappa }}^{\mathrm{1D}}+i\hat{\omega }}\right)}{\tau \hat{L}\sqrt{\langle \alpha_{0}\rangle {\hat{\kappa }}^{\mathrm{1D}}+i\hat{\omega }}}.$$ (25)

Thus, the expression for calculating the effective tortuosity can be modified as

$$\left|\mathrm{\tau coth}\left(\tau \hat{L}\sqrt{\langle \alpha_{0}\rangle {\hat{\kappa }}^{\mathrm{1D}}+i\hat{\omega }}\right)\right|=\left|\frac{Z^{\mathrm{3D}}}{Z^{\mathrm{FLG}}}\left(\left(\mathrm{1-ɛ}\right)\coth\left(\hat{L}\sqrt{\langle \alpha_{0}\rangle {\hat{\kappa }}^{\mathrm{1D}}+i\hat{\omega }}\right)\right)\right|.$$ (26)

### Simulated Impedance and Tortuosity Calculation

**FIGURE 5:**
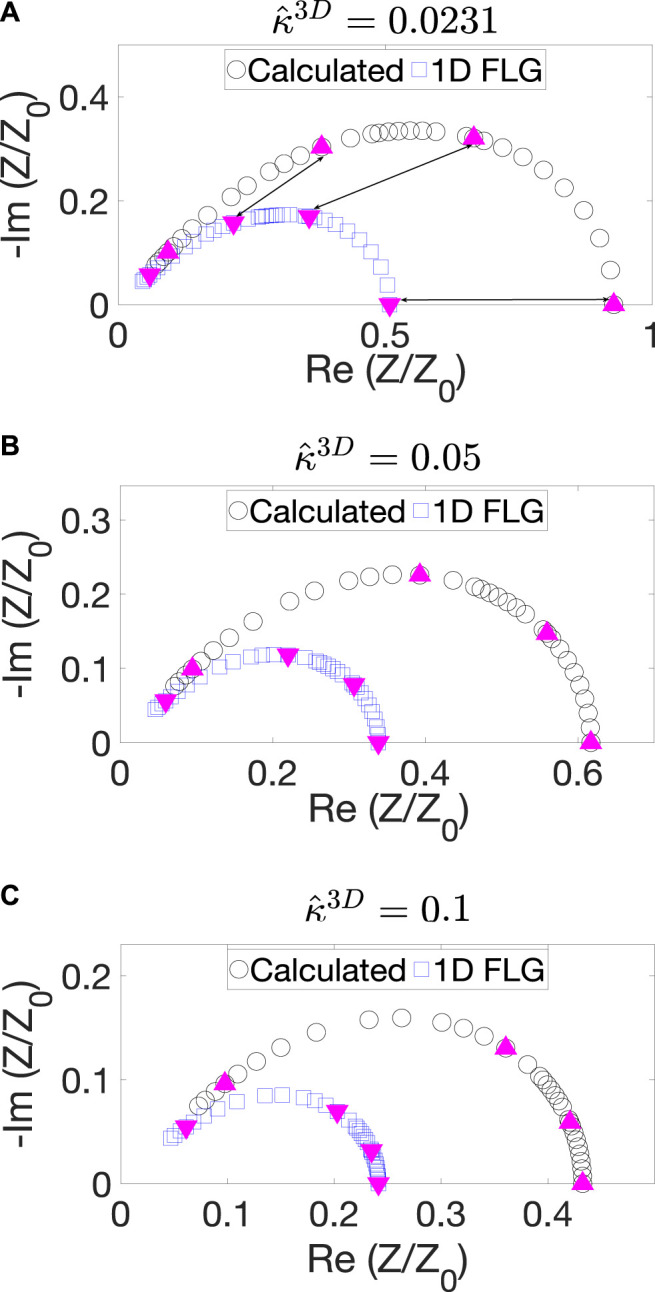
Nyquist plots obtained from 3D calculations for the BCC BC (black curve) and 1D FLG model (blue curve) for **(A)** ${\hat{\kappa }}^{3D}=0.0231$, **(B)**  0.05, and **(C)**  0.1. The iso-frequency points are marked with upright magenta triangles on the black curves and inverted magenta triangles on the blue curves. Three iso-frequency points are connected with arrows in a).

Figure 5 shows the impedance spectra of the experimentally obtained microstructure for three values of ${\hat{\kappa }}^{3D}$, along with the impedance spectra obtained from the analytical expression for the 1D FLG impedance in Eq. 18. As expected, the two curves for the same value of ${\hat{\kappa }}^{3D}$ deviate significantly, with the FLG curve underestimating the impedance value. To enable a better visualization of the difference a few iso-frequency points, on both the curves, are marked with magenta triangles. We note that the discussion below is for a fixed cathode thickness provided in the numerical implementation section. The effect of cathode thickness on the impedance and tortuosity is discussed qualitatively in Supplementary Material.

**FIGURE 6:**
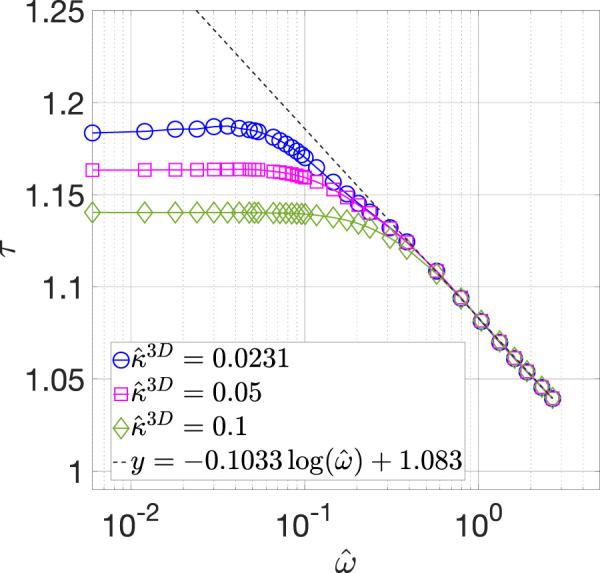
Calculated τ vs. $\hat{\omega }$ relations (BCC BC) for ${\hat{\kappa }}^{3D}=0.0231$ (blue circles), 0.05 (magenta squares), and 0.1 (green diamonds). The black dashed line represents the fit with a linear function at high frequencies.

By solving Eq. 26, we calculated τ for the three values of ${\hat{\kappa }}^{3D}$ over the entire frequency range examined. The results are shown in Figure 6. From this comparison, three trends can be observed in the tortuosity data. First, for a given value of ${\hat{\kappa }}^{3D}$,  τ remains almost a constant before it begins to decrease with an increase in $\hat{\omega }$. The relation between τ and $\log\left(\hat{\omega }\right)$ at high frequencies can be represented by a linear function with a slope of −0.1033, as shown by black dashed line in Figure 6. Second, the low frequency value of τ decreases with an increase in ${\hat{\kappa }}^{3D}$. Finally, all three τ vs. $\hat{\omega }$ curves coincide at high frequencies.

**FIGURE 7:**
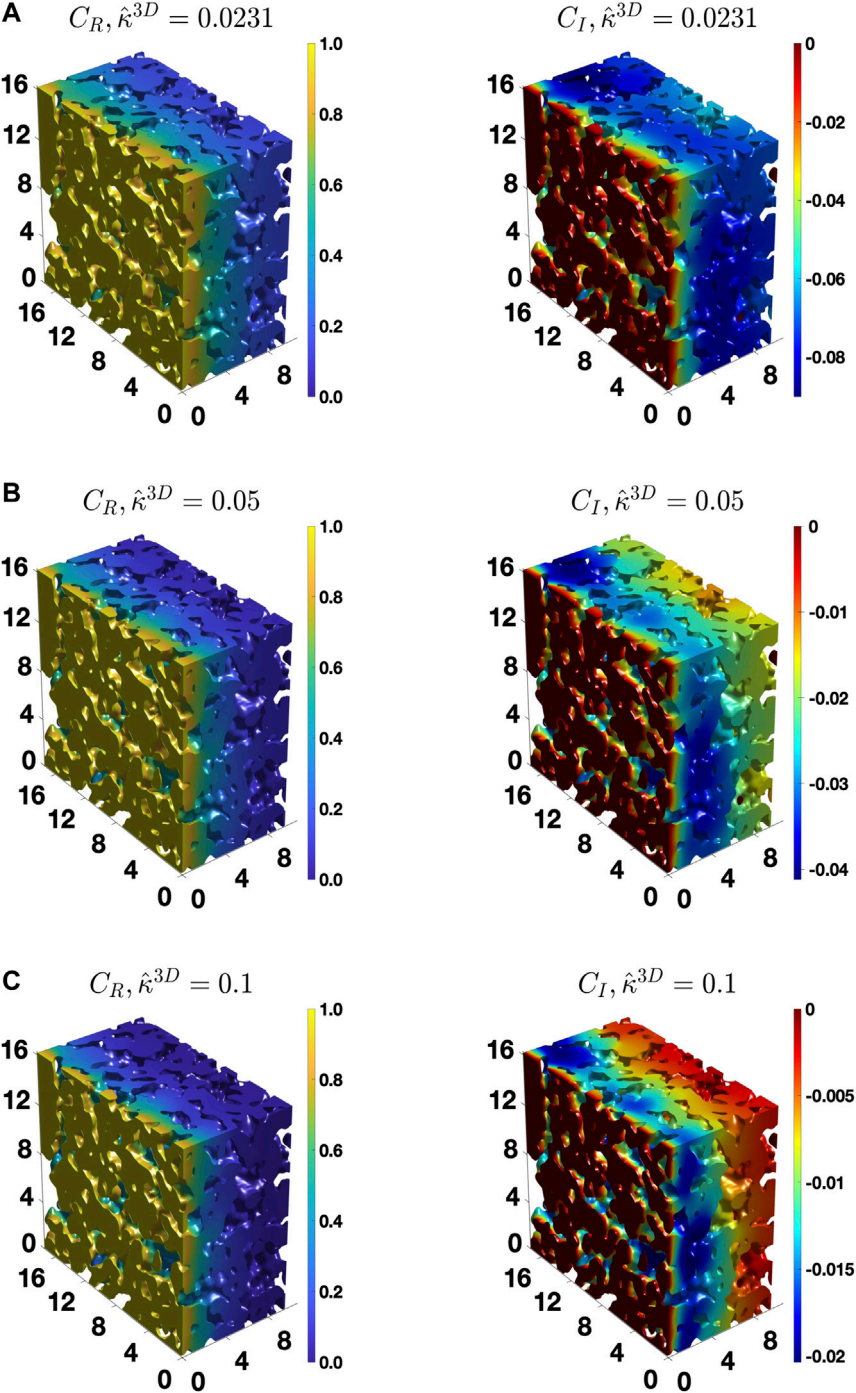
The distribution of the real and imaginary components of the concentration amplitude calculated for the BCC BC, $\hat{\omega }\;=\;0.018$ and **(A)** ${\hat{\kappa }}^{3D}\;=\;0.0231$, **(B)** 0.05, and **(C)**  0.1.

**FIGURE 8:**
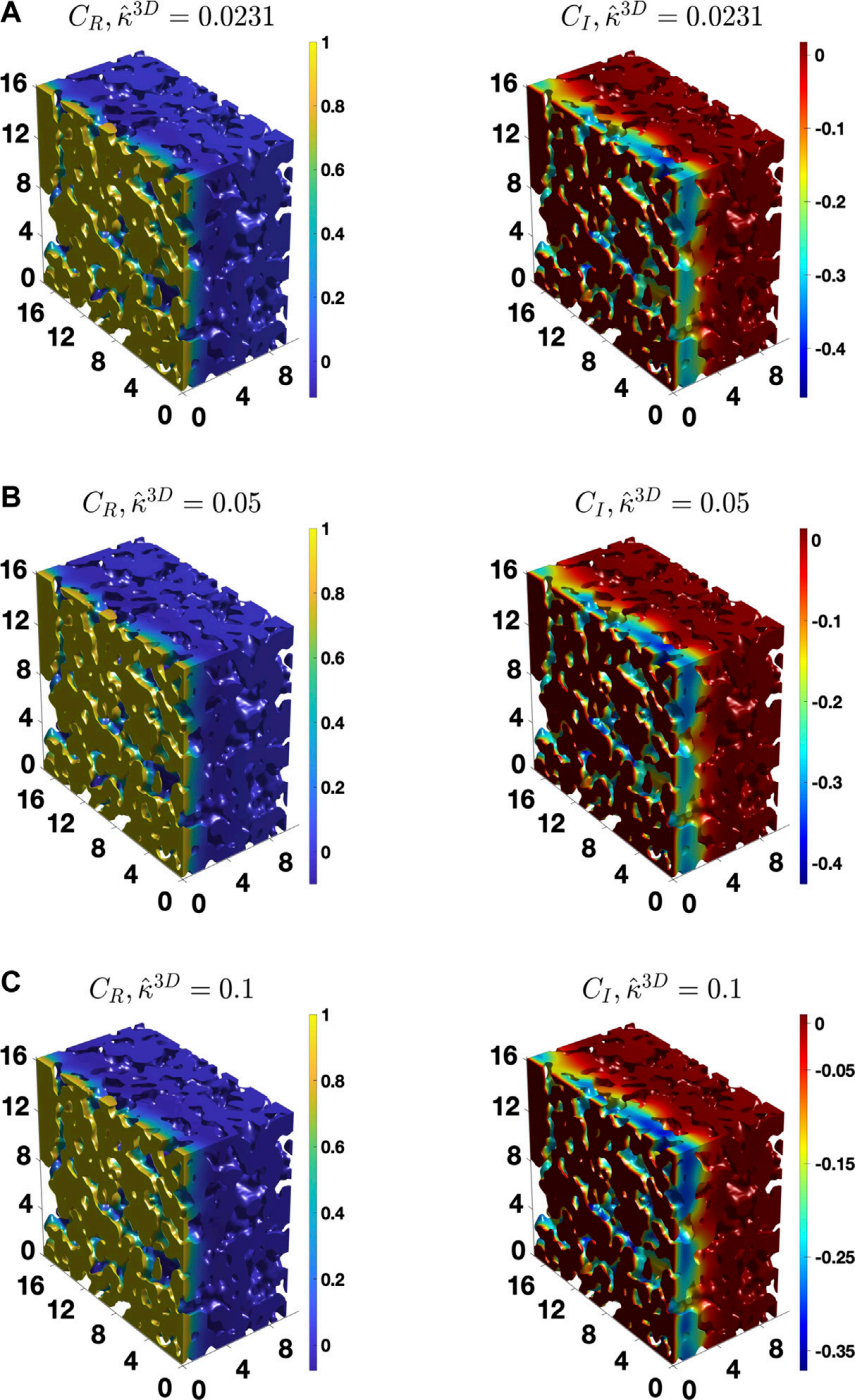
The distribution of the real and imaginary components of the concentration amplitude calculated for the BCC BC, $\hat{\omega }\;=\;1.038$ and **(A)** ${\hat{\kappa }}^{3D}\;=\;0.0231$, **(B)** 0.05, and **(C)** 0.1.

To provide further insights, Figure 7 shows the distribution of $C_{R}$ and $C_{I}$ in the complex microstructure at a low value of $\hat{\omega }$, 0.018, which is close to the DC case, for three different ${\hat{\kappa }}^{3D}$ values. For the low value of ${\hat{\kappa }}^{3D}$, 0.0231, the gradient in the concentration amplitude is non-zero in much of the microstructure thickness, as shown in Figure 7A. However, with an increase in the value of ${\hat{\kappa }}^{3D}$, the gradient spans lesser and lesser of the microstructure at the same frequency, as qualitatively shown in Figures 7B,C. Thus, it is evident that the penetration depth of the diffusing species (oxygen vacancies) at the same frequency of the AC load decreases with an increase in ${\hat{\kappa }}^{3D}$. Furthermore, Figure 8 shows the distribution of $C_{R}$ and $C_{I}$ in the microstructure for three values of ${\hat{\kappa }}^{3D}$ at a higher frequency value, $\hat{\omega }\;=\;1.038$. As can be seen, the gradient in the concentration amplitude is mostly confined to a small region near the electrolyte/cathode interface for all values of ${\hat{\kappa }}^{3D}$ at the high frequency regime. Therefore, the penetration depth for each case at the high frequency value is much smaller than at the low frequency value. Moreover, at $\hat{\omega }\;=\;1.038$ the penetration depth is similar for all three values of ${\hat{\kappa }}^{3D}$. This shows that the penetration depth is a function of both ${\hat{\kappa }}^{3D}$ and $\hat{\omega }$. Since the effective tortuosity of a microstructure is directly influenced by the penetration depth (Yu et al., 2020), it is evident that the effective tortuosity is also a function of both ${\hat{\kappa }}^{3D}$ and $\hat{\omega }$. Therefore, it can be inferred that, for a porous medium, where the rate kinetics is co-limited by both the bulk diffusion and surface reaction, the tortuosity is a function not only of the microstructural characteristics but also of ${\hat{\kappa }}^{3D}$, which is a combination of material property. In addition, it also depends on the frequency, as was found in the case for diffusional impedance case (Yu et al., 2020). The observed behavior of the effective tortuosity could also be observed from the distribution of reaction-diffusion streamlines (Zhang Y. et al., 2019), which is a 3D visualization of the flux. As the penetration depth decreases, the streamlines or the trajectories of the oxygen vacancies begin to straighten. The resulting decrease in the deviation from a straight path causes the effective tortuosity to decrease.

We note that there exists an error of up to 9% in the calculated impedance value at high frequencies, as described in Supplementary Material. The error arises because the accuracy of numerical approximations of the gradients in the concentration amplitudes decreases at high frequencies, as our numerical implementation employs fixed grid resolution throughout the simulation domain. Although this error can be reduced by doubling the grid resolution, such calculations become computationally expensive (as discussed in Supplementary Material) without providing additional insights. In fact, the error does not affect the observed qualitative behavior of the effective tortuosity, which is the one of the two main focuses of this work. Thus, the numerical results presented here are sufficiently accurate for demonstrating the dependence of the effective tortuosity on ${\hat{\kappa }}^{3D}$ and $\hat{\omega }$. In the future, we plan to implement the model into a finite element framework with adaptive-mesh capability such as PRISMS-PF framework (Aagesen et al., 2018; DeWitt et al., 2020), in which SBM is already implemented, to increase the computational efficiency of the calculations at high frequencies.

**FIGURE 9:**
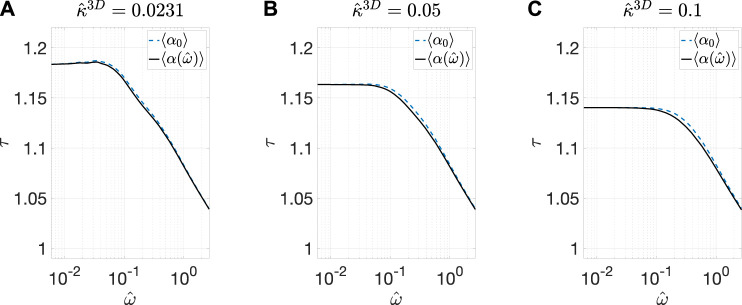
Comparisons of the calculated τ vs. $\hat{\omega }$ relations (BCC BC) by using 〈$\alpha_{0}$〉 (blue curve) and 〈α〉 (black curve) for **(A)** ${\hat{\kappa }}^{3D}\;=\;0.0231$, **(B)** 0.05, and **(C)**  0.1.

Finally, to further evaluate the accuracy of approximating 〈α〉 with 〈$\alpha_{0}$〉 in the modified ALS model, we compared the tortuosity results with and without this approximation, latter of which include the frequency dependence of 〈α〉. A comparison between the two sets of τ value obtained for the three values of ${\hat{\kappa }}^{3D}$ is provided in Figure 9, which show good agreement. Hence the use of 〈$\alpha_{0}$〉 as the correction factor is sufficiently accurate for calculating the effective tortuosity of a MIEC SOFC cathode using the EIS data.

## Summary

In this study, we simulated the impedance behavior of a statistically representative portion of an experimentally determined complex three-dimensional microstructure of an unbiased MIEC SOFC cathode under two different boundary conditions. Our investigation generated two key insights. First, due to the presence of surface reaction, the amplitude of oscillations in the vacancy concentration is lower at the pore/solid interface than within the solid bulk of the cathode. This difference between the interface and bulk amplitude increases with an increase in the ratio between the reaction rate constant and the bulk diffusion coefficient. Therefore, to account for this difference, we proposed a modification to the ALS model in terms of the ratio of the surface and bulk concentration amplitudes at zero frequency, 〈$\alpha_{0}$〉, and provided numerically evaluated 〈$\alpha_{0}$〉 as a function of the reaction rate coefficient, ${\hat{\kappa }}^{3D}$. Second, through the examination of the three-dimensional distribution of the vacancy concentration amplitude, we showed that the penetration depth of the oxygen vacancies is a function of ${\hat{\kappa }}^{3D}$ and the frequency of the applied AC load, $\hat{\omega }$. Due to the direct dependence of the effective tortuosity on the penetration depth, the effective tortuosity also becomes a function of ${\hat{\kappa }}^{3D}$ and $\hat{\omega }$ in addition to the microstructure. Furthermore, we developed a method, which utilizes the EIS data, for determining τ as a function of ${\hat{\kappa }}^{3D}$ and $\hat{\omega }$ for a cathode with known material properties (such as the reaction rate constant and bulk diffusion constant), pore surface area, and porosity.

## Data Availability

The data for all the figures in this paper can be downloaded from the following link: https://doi.org/10.13011/m3-ywxf-yw20.
