# Supplementary material for: Simulation of the Electrochemical Impedance in a Three-Dimensional, Complex Microstructure of Solid Oxide Fuel Cell Cathode and Its Application in the Microstructure Characterization
Source: Front Chem. 2021 May 27;9:627699. doi: 10.3389/fchem.2021.627699 (PMC8190658; doi:10.3389/fchem.2021.627699)
Supplement: Supplementary file 1 [file DataSheet1.pdf]

Simulation of the electrochemical impedance in a three-dimensional, complex microstructure of solid oxide fuel cell cathode and its application in the microstructure characterization

Vishwas Goel[1], Dalton Cox[2], Scott A. Barnett[2], Katsuyo Thornton[1,*]
[1] Department of Materials Science and Engineering, University of Michigan, Ann Arbor, MI, USA
[2] Department of Materials Science and Engineering, Northwestern University, Evanston, IL, USA

*Correspondence:
Katsuyo Thornton
kthorn@umich.edu

Supplementary Material

Numerical accuracy analysis for the SBM grid

We tested the grid spacing used in the three-dimensional calculations by considering a model cylindrical microstructure of a unit (non-dimensional) length and radius. These dimensions were selected to ensure that the cylinder has the same surface area to volume ratio as the complex microstructure; such a selection enables us to assume a similar numerical behavior of our calculations involving the experimental microstructure as those involving the model cylindrical microstructure.

Two grid related parameters affect the accuracy of the SBM-based calculations, namely, the grid spacing h and interfacial thickness λ. We tested three value pairs of the grid parameters. First, keeping the parameter values to be same as the complex microstructure ($h = 0.0272$, $\lambda = 0.0817$), second, decreasing the grid spacing to half and retaining the same interfacial thickness, and third, decreasing both the grid spacing and interfacial thickness to half of the first set of values. It should be noted that among the three configurations, third is the most refined grid while first is the least refined grid. We compare the calculated impedance values for the three configurations at three frequency values $\hat{\omega} = 1.038$, 42, and 95, and the results are summarized in Supplementary Table 1 along with the % difference in the real and imaginary components of the impedance with respect to results for the third configuration.

For the lowest frequency case, which is comparable to $\hat{\omega} = 0.011$ case in the simulations with the experimental microstructure, the error is small in both the calculated concentration amplitudes and the impedance. In Supplementary Figure 1a, C_R and C_I are shown for the test cases for this frequency. The maximum differences in in C_R, C_I, and the impedance values between the first and third configuration is ~ 0.1%, 1%, and 1% for this frequency.

For the intermediate frequency case, which is comparable to $\hat{\omega} = 0.46$ case in the simulations with the experimental microstructure, the error in C_R and C_I is of the same order as that of the previous case, as shown in Supplementary Figure 1b. However, the error in the real component of the

impedance value increased to 5.75% while that in the imaginary component remains less than 1%, as shown in Supplementary Table 1. A similar trend in errors is observed for the high frequency case, which is comparable to $\hat{\omega} = 1.038$ case in the simulations with the experimental microstructure. The error in C_R and C_I is still comparable to the low and intermediate frequency cases. However, the error in the real component of the impedance value increased to 8.68% while that in the imaginary component remains less than 1%, as shown in Supplementary Table 1.

From the above analysis, it can be concluded that the first grid configuration ($h = 0.0272,\ \lambda = 0.0817$) is sufficiently refined to accurately calculate the value of C_R and C_I (and therefore $\langle \alpha \rangle$) in the cylindrical microstructure, and consequently, in the experimental microstructure over the entire frequency range studied. However, the numerical accuracy of the first grid configuration for the impedance calculation starts decreasing at intermediate to high frequency values. This decrease in accuracy is expected because with increasing frequency, the gradients in the concentration amplitude becomes larger in magnitude, which becomes harder to resolve with a fixed grid resolution. Nonetheless, the maximum error observed in the impedance value is ~ 9%, which is acceptable because of two reasons. First, this error only occurs at high frequency values and the error leads to an overestimation of the impedance value. Thus, the error does not alter the qualitative behavior of the calculated tortuosity, which is the one of two main focuses of this paper. Second, the selected resolution offers a good balance between accuracy and computational efficiency. Although the error in the impedance values at high frequencies can be reduced by doubling the grid resolution, a simulation with such a grid would be computationally expensive without yielding additional insights. With the current grid configuration, a typical impedance calculation for the complex microstructure at a given frequency takes ~12.5 hrs on 288 CPU cores. Doubling the resolution would result in an eightfold increase in the system size, and thus in the computation time, which makes it costly to run, especially if multiple materials parameters must be examined.

In conclusion, the selected grid parameters ($h = 0.0272,\ \lambda = 0.0817$) offers high accuracy in the calculation of C_R and C_I over all frequency values and in the calculation of impedance at low frequency values. For intermediate to high frequency values, this resolution results in up to 9% overestimation in the impedance value; however, the additional accuracy would not lead to new insights for the phenomena considered in this paper. If quantitative predictions at the high frequency regime is desired, higher resolution, possibly with adaptive mesh, should be considered.

Supplementary Table 1. List of the errors in the real and imaginary components of impedance calculated for three different grid.

$\hat{\omega}$	h	λ	Z^{3D}/Z_0	% difference in real component	% difference in imaginary component
1.038	0.0272	0.0817	3.31 – 4.52i	0.91%	1.1%
	0.0136	0.0817	3.27 – 4.47i	0.30%	0.04%
	0.0136	0.0408	3.28 – 4.47i	--	--
42	0.0272	0.0817	0.625 – 0.550i	5.75%	0.36%
	0.0136	0.0817	0.591 – 0.552i	0.02%	0.02%
	0.0136	0.0408	0.591 – 0.552i	--	--
95	0.0272	0.0817	0.438 – 0.366i	8.68%	0.60%

	0.0136	0.0817	0.403 – 0.368i	0.02%	0.03%
	0.0136	0.0408	0.403 – 0.368i	--	--

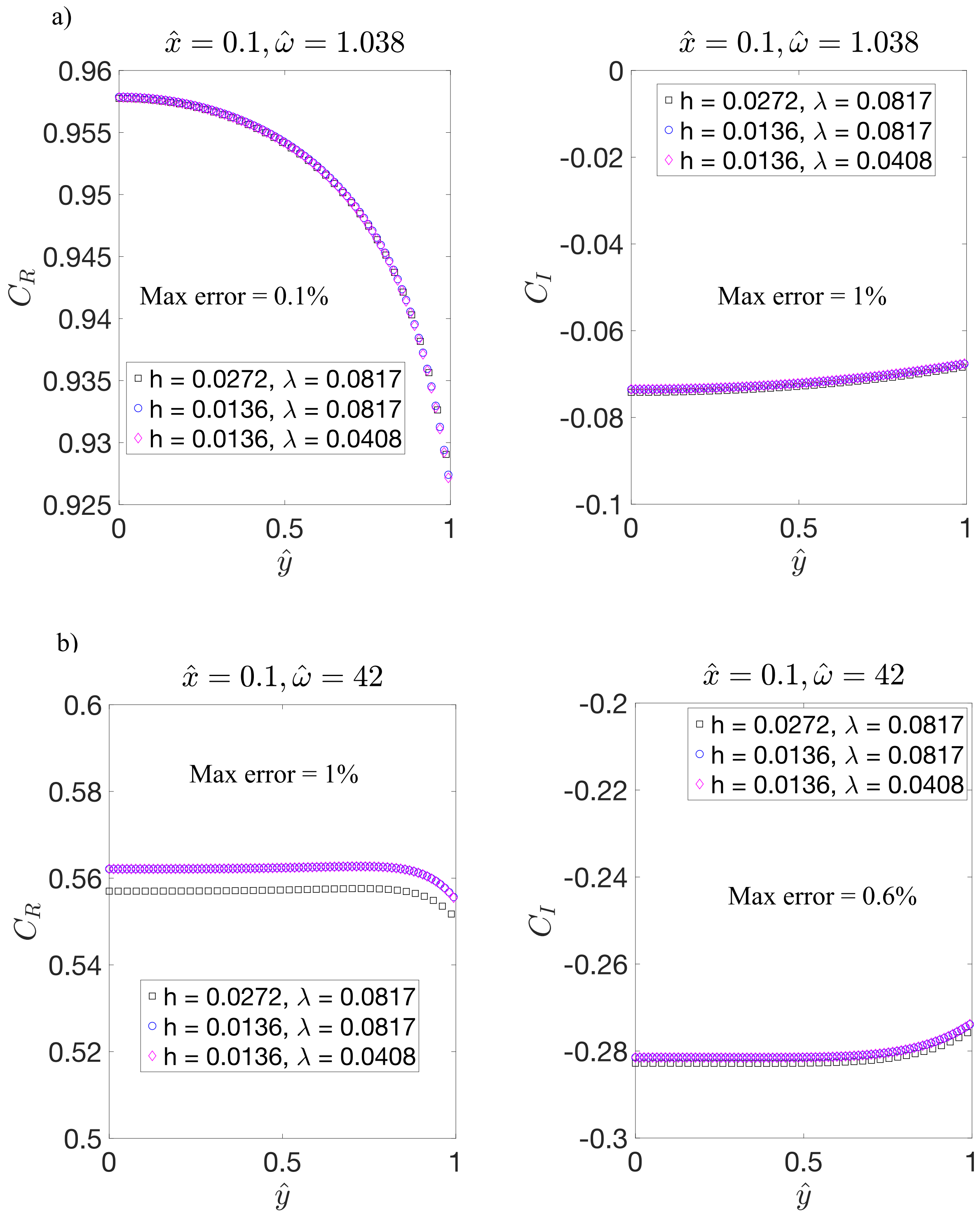

c)

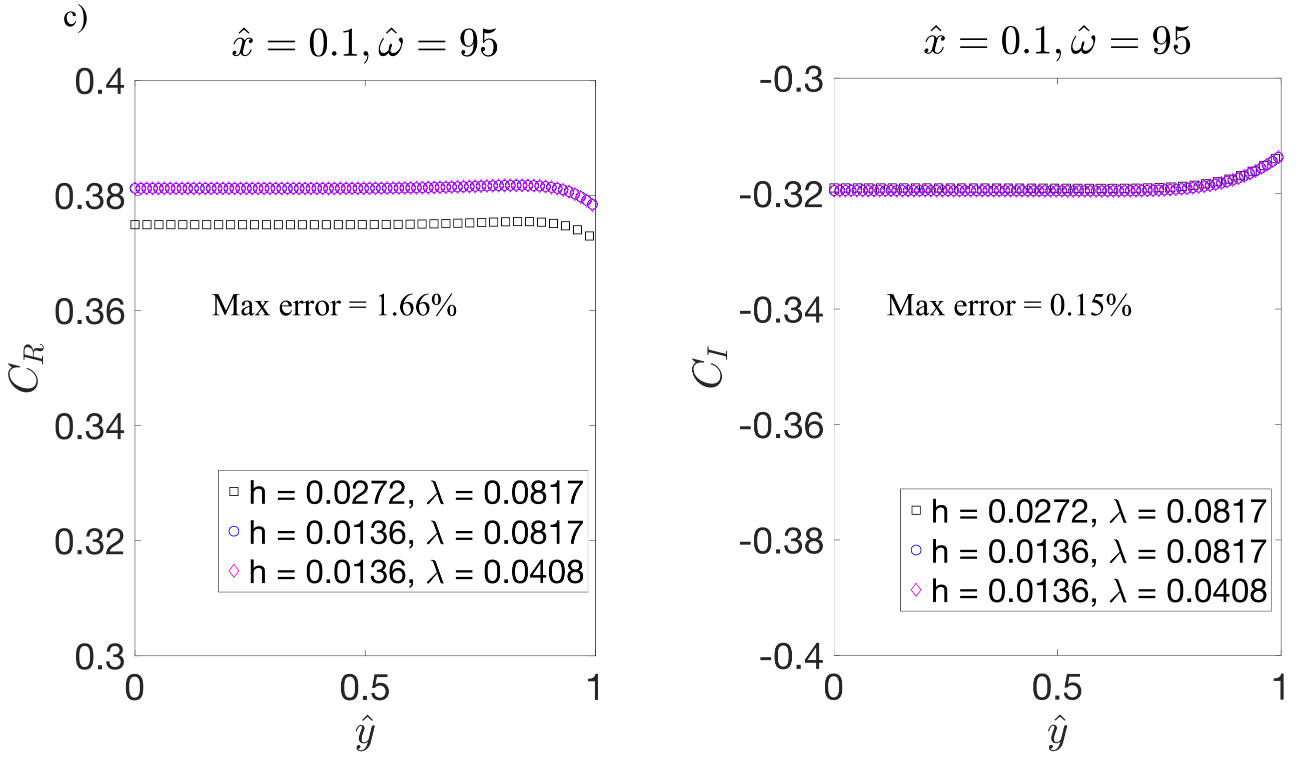

Supplementary Figure 1. Plot of C_R and C_I as a function of cylinder radius for the three grid configurations at $\hat{x} = 0.1$ and a) $\widehat{\omega} = 1.038$, b) $\widehat{\omega} = 42$, and c) $\widehat{\omega} = 95$.

Effect of cathode thickness on the impedance

The effect of cathode thickness on the impedance can be predicted by the modified ALS model, Eq. 25. By taking Z_0 on the right hand side, it can be seen that there is the impedance value has an explicit dependence on the cathode thickness in terms of the $coth$ term, and an implicit dependence in terms of tortuosity. To analyze this dependence, we consider cathodes of the same material with three different thicknesses under a DC load.

First, we consider a cathode thickness that is much larger than the penetration depth. For such a case, the impedance value does not change with a change in the cathode thickness assuming that there is no gas transport limitation. This is because at a such large cathode thickness, the $coth$ term is almost equal to 1 while tortuosity remains equal to the value corresponding to the penetration depth. Second, we consider a cathode thickness that is smaller than the penetration depth but larger than the average feature size of the microstructure (solid volume/surface area). For such a case, if the cathode thickness is decreased, a decrease in tortuosity will also be observed. However, the $coth$ term will increase much more rapidly because of its decreasing argument. Therefore, the

cathode impedance will increase as the cathode thickness is increased. Finally, we consider a cathode thickness that is sufficiently smaller than the average feature size of the microstructure. For this case, tortuosity will be ~ 1 because the microstructural features are truncated by the current-collector/cathode and electrolyte/cathode interfaces and oxygen vacancies can flow directly from one boundary to the other boundary in a straight path. However, the $coth$ term will continue to increase with a reduction in cathode thickness. Therefore, for this case, any further reduction in cathode thickness will lead to an increase in the cathode impedance.

Results for the TCC BC

The results for the TCC BC are provided here.

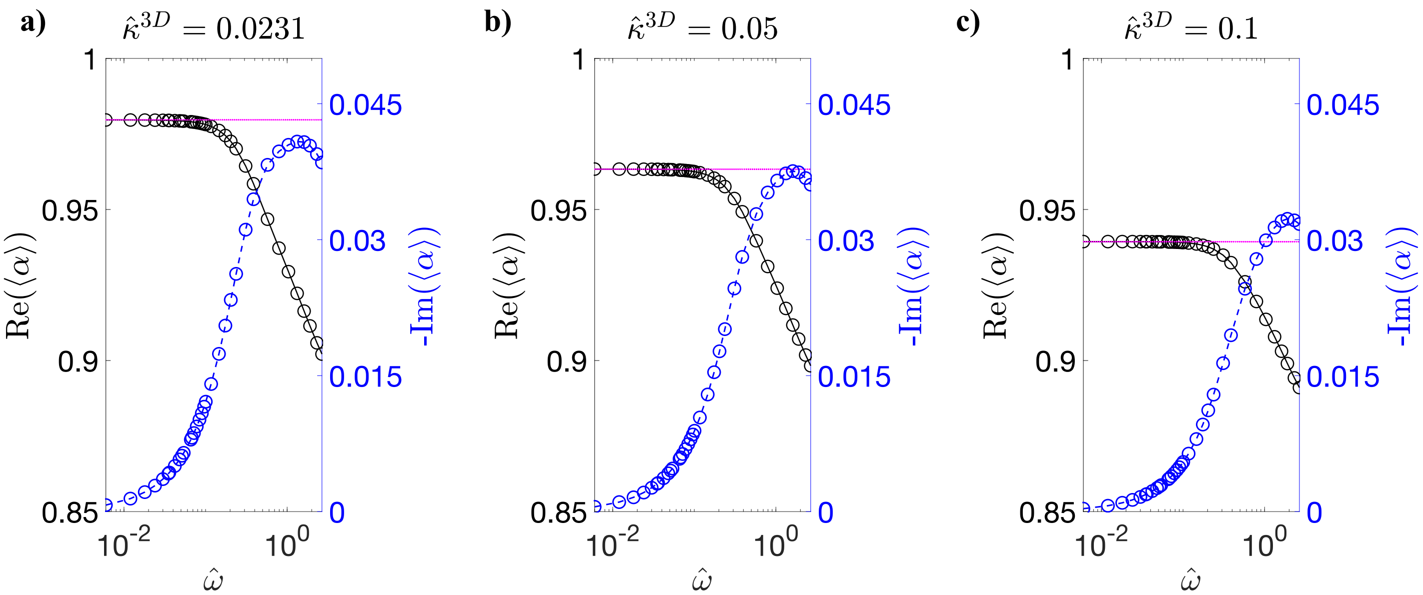

Supplementary Figure 2. Calculated $\langle\alpha\rangle$ vs. $\widehat{\omega}$ (TCC BC) at a) $\hat{\kappa}^{3D} = 0.0231$, b) 0.05, and c) 0.1. The real component (black solid curve) and the imaginary component (blue dashed curve) of $\langle\alpha\rangle$ are plotted on the left and right y-axes, respectively. The calculated points are noted by circles. The DC values of $\langle\alpha\rangle$ are shown with horizontal magenta dotted lines as a reference.

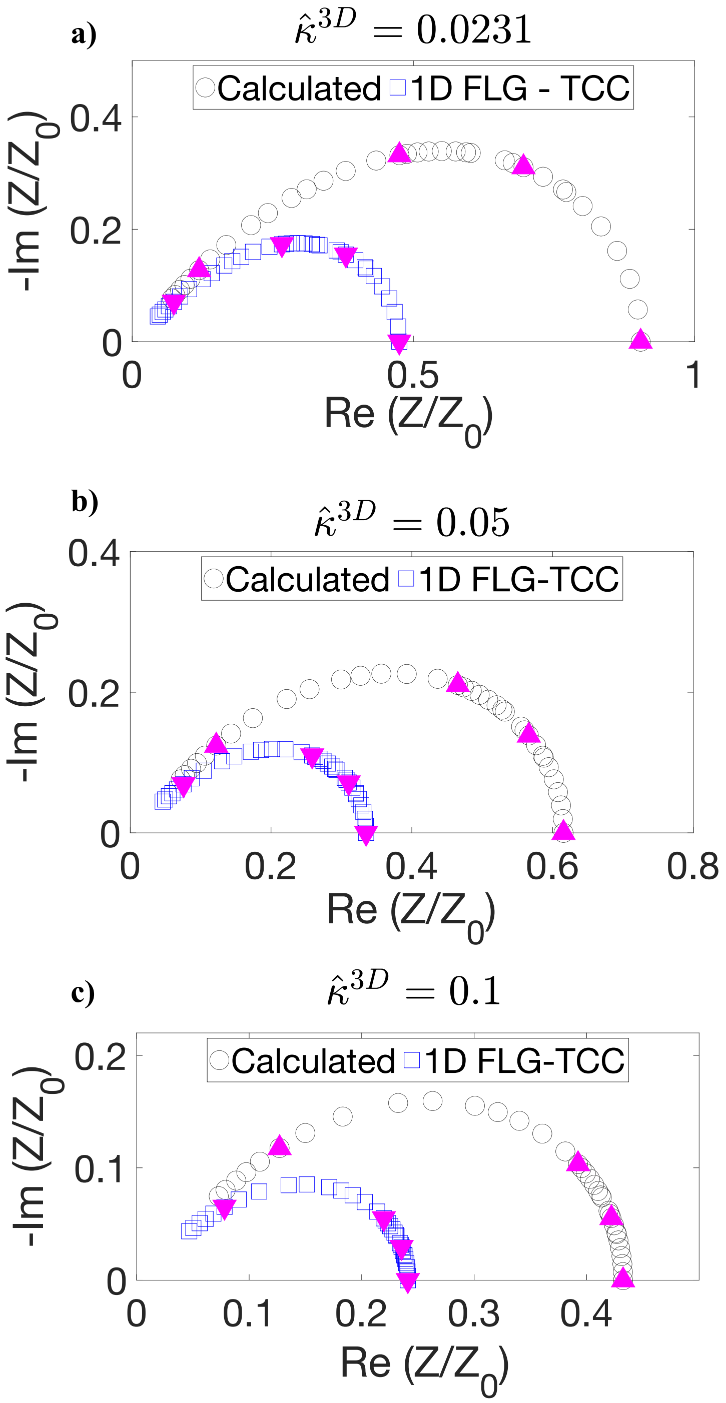

Supplementary Figure 3. Nyquist plots obtained from 3D calculations for the TCC BC (black circles) and 1D FLG-TCC model (blue squares) for a) $\hat{\kappa}^{3D} = 0.0231$, b) 0.05, and c) 0.1. The iso-frequency points are marked with upright magenta triangles on the black circles and inverted magenta triangles on the blue curves.

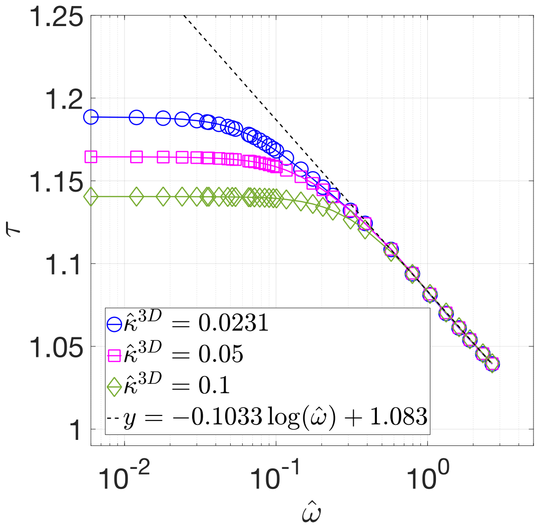

Supplementary Figure 4. Calculated τ vs. $\widehat{\omega}$ relations (TCC BC) for $\hat{\kappa}^{3D} = 0.0231$ (blue circles), 0.05 (magenta squares), and 0.1 (green diamonds). The black dashed line represents the fit with a linear function at high frequencies.

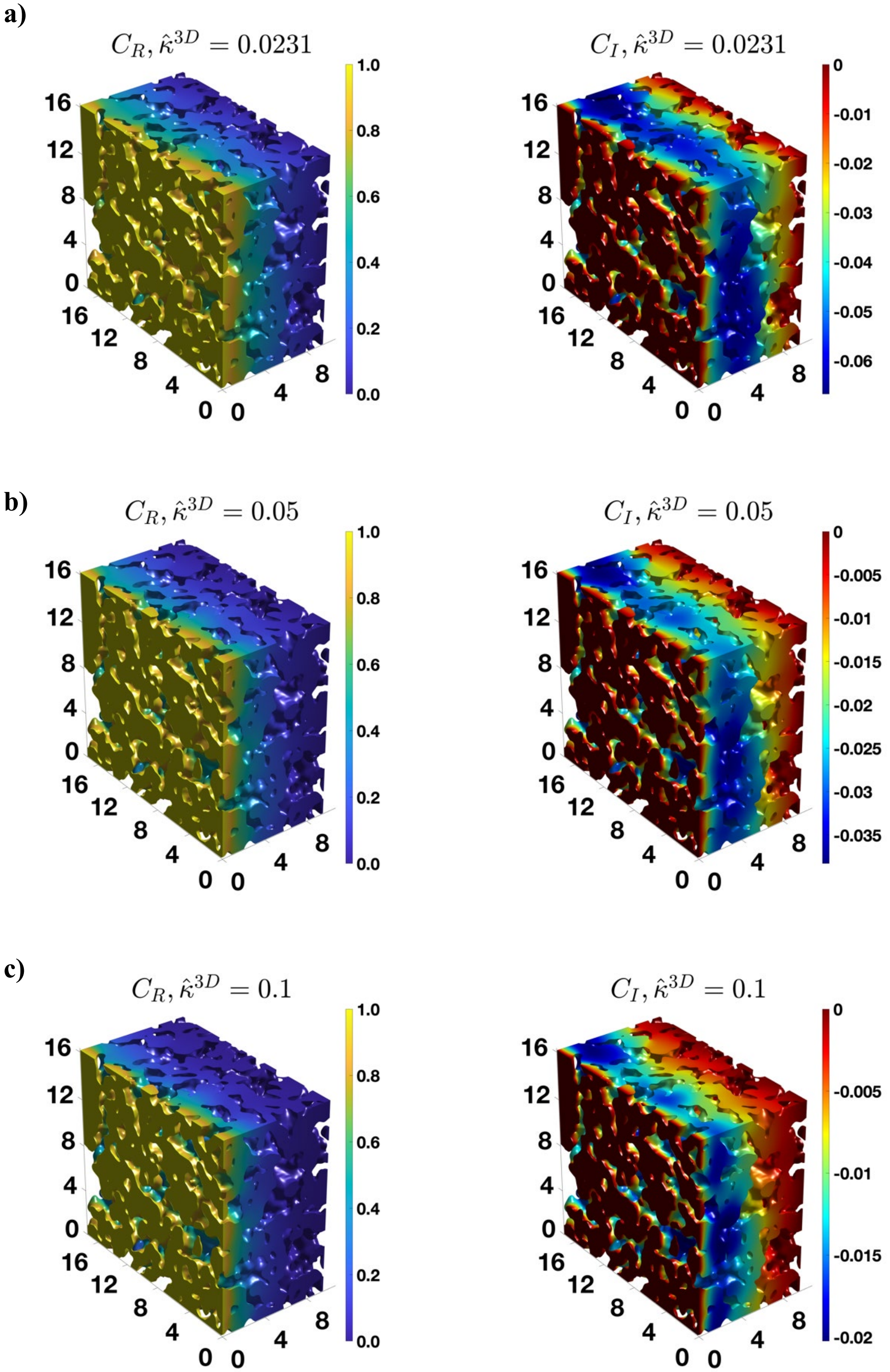

Supplementary Figure 5. The distribution of the real and imaginary components of the concentration amplitude calculated for the TCC BC, $\hat{\omega} = 0.018$ and a) $\hat{\kappa}^{3D} = 0.0231$, b) 0.05, and c) 0.1.

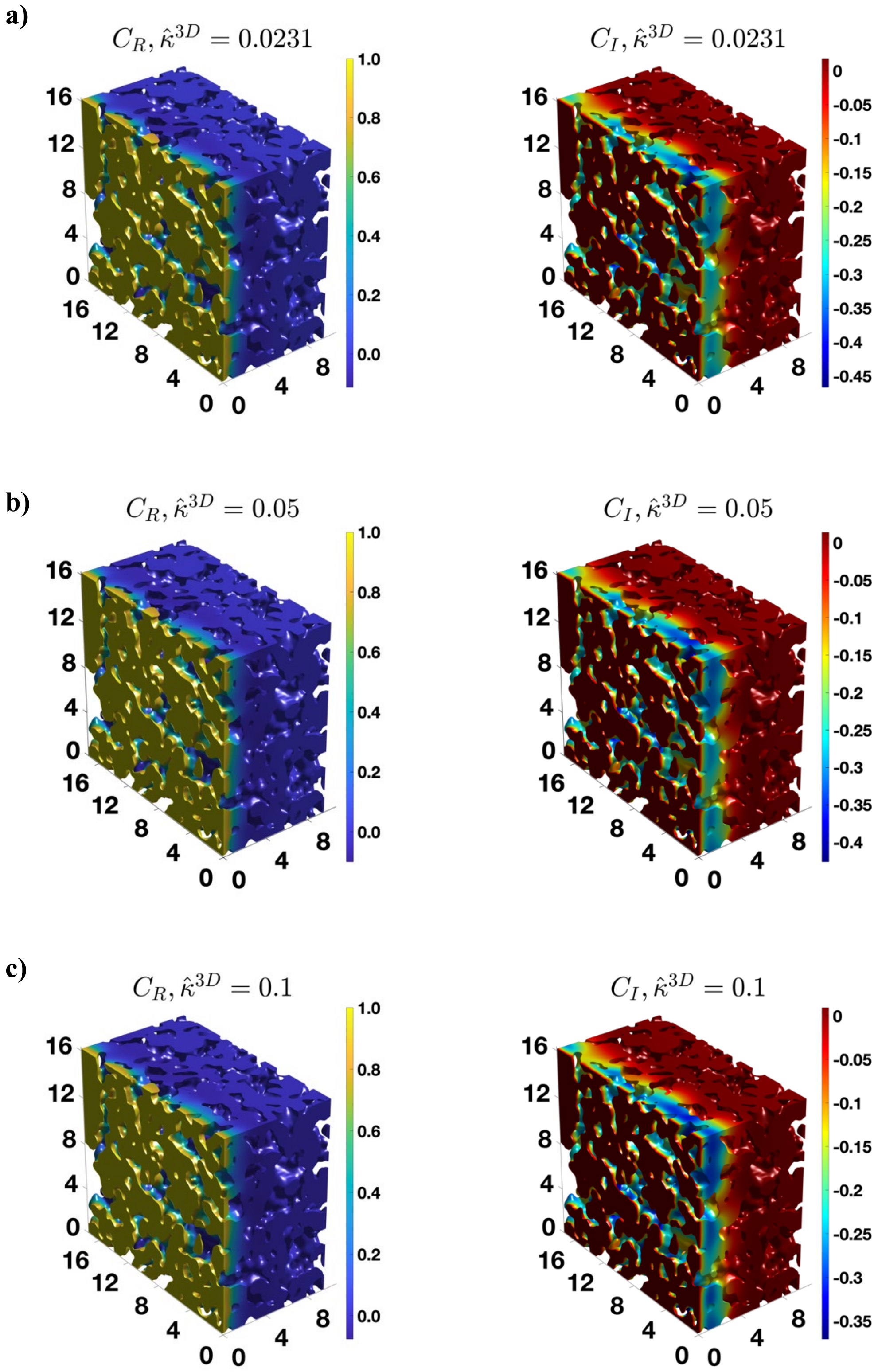

Supplementary Figure 6. The distribution of the real and imaginary components of the concentration amplitude calculated for the TCC BC, $\hat{\omega} = 1.038$ and a) $\hat{\kappa}^{3D} = 0.0231$, b) 0.05, and c) 0.1.

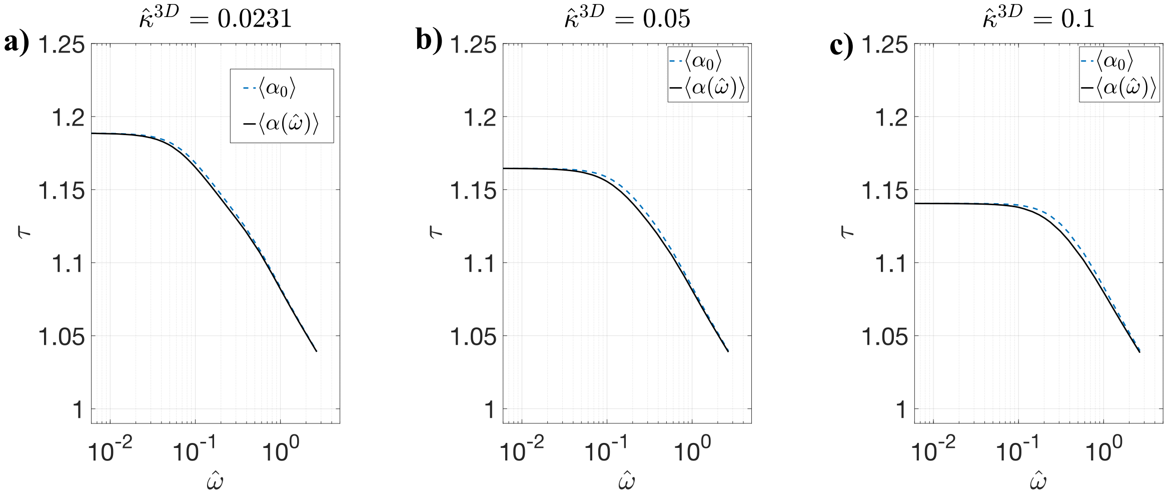

Supplementary Figure 7. Comparison of calculated τ vs. $\widehat{\omega}$ relations (TCC BC) by using $\langle\alpha_0\rangle$ (blue curve) and $\langle\alpha\rangle$ (black curve) for a) $\hat{\kappa}^{3D} = 0.0231$, b) 0.05, and c) 0.1.
